# Kinin B_2_ and B_1_ Receptors Activation Sensitize the TRPA1 Channel Contributing to Anastrozole-Induced Pain Symptoms

**DOI:** 10.3390/pharmaceutics15041136

**Published:** 2023-04-03

**Authors:** Maria Fernanda Pessano Fialho, Evelyne Silva Brum, Gabriela Becker, Indiara Brusco, Sara Marchesan Oliveira

**Affiliations:** 1 Graduate Program in Biological Sciences, Biochemical Toxicology, Center of Natural and Exact Sciences, Federal University of Santa Maria, Camobi, Santa Maria 97105-900, RS, Brazil; 2 Department of Biochemical and Molecular Biology, Center of Natural and Exact Sciences, Federal University of Santa Maria, Camobi, Santa Maria 97105-900, RS, Brazil

**Keywords:** nociception, muscle strength loss, musculoskeletal pain, adjuvant endocrine therapy, PLC/PKC pathways

## Abstract

Aromatase inhibitors (AIs) cause symptoms of musculoskeletal pain, and some mechanisms have been proposed to explain them. However, signaling pathways downstream from kinin B_2_ (B_2_R) and B_1_ (B_1_R) receptor activation and their possible sensitizing of the Transient Receptor Potential Ankyrin 1 (TRPA1) remain unknown. The interaction between the kinin receptor and the TRPA1 channel in male C57BL/6 mice treated with anastrozole (an AI) was evaluated. PLC/PKC and PKA inhibitors were used to evaluate the signaling pathways downstream from B_2_R and B_1_R activation and their effect on TRPA1 sensitization. Anastrozole caused mechanical allodynia and muscle strength loss in mice. B_2_R (Bradykinin), B_1_R (DABk), or TRPA1 (AITC) agonists induced overt nociceptive behavior and enhanced and prolonged the painful parameters in anastrozole-treated mice. All painful symptoms were reduced by B_2_R (Icatibant), B_1_R (DALBk), or TRPA1 (A967079) antagonists. We observed the interaction between B_2_R, B_1_R, and the TRPA1 channel in anastrozole-induced musculoskeletal pain, which was dependent on the activation of the PLC/PKC and PKA signaling pathways. TRPA1 seems to be sensitized by mechanisms dependent on the activation of PLC/PKC, and PKA due to kinin receptors stimulation in anastrozole-treated animals. Thus, regulating this signaling pathway could contribute to alleviating AIs-related pain symptoms, patients’ adherence to therapy, and disease control.

## 1. Introduction

Cancer incidence is increasing each year, causing high morbidity and mortality rates. Breast cancer is the most commonly diagnosed cancer, with an estimated more than 2.3 million new cases (11.7%) per year [1]. Most breast cancer survivors carry a burden of sequelae after diagnosis and treatment, with pain being one of the most common symptoms [1,2]. The causes of pain are varied and might be related to the adjuvant endocrine treatment prescribed after the cancer treatment [2,3].

The third-generation aromatase inhibitors (AIs) are considered first-line drugs in the adjuvant endocrine treatment for postmenopausal women diagnosed with hormone receptor-positive breast cancer, and the most prescribed is anastrozole, followed by letrozole and exemestane [4,5,6]. The AIs are recommended for at least five years after diagnosis or may be used after therapy with tamoxifen (subsequent 2–3 years) [3,5,7]. One-third of AIs users have reported adverse effects from treatment onset [6,8,9]. Painful musculoskeletal symptoms, characterized by morning stiffness, joint pain, myalgia, and decreased strength, seem to be preponderant daily symptoms in these patients [3,8]. Furthermore, this painful condition can include diffuse (22%), mixed (11%), or neuropathic (9%) pain [8,9,10]. The persistent symptoms significantly impact patients’ quality of life and treatment compliance, leading to at least a quarter of patients discontinuing therapy [8].

The genesis of AIs-related pain is unclear, but it can be attributed to factors such as oestrogen deprivation, previous hormone therapy and/or taxane-based chemotherapy, pre-existing arthralgia, or a surgical procedure after a cancer diagnosis (mastectomy) [3,9,10,11,12]. Therapeutic approaches that mitigate pain associated with inflammatory joint disorders are ineffective at alleviating AIs-induced pain [10]. Thus, elucidating this condition’s underlying mechanisms and pathways in experimental models may have translational potential for developing novel targets to treat the pain symptoms associated with AIs use.

Some mechanisms have been proposed to explain AIs-related pain symptoms, among them the altered function of ion channels in sensory neurons, such as Transient Potential Receptor Ankyrin 1 (TRPA1) [13]. Indeed, the TRPA1 channel is emerging as one of the main mediators in several pain models, such as those induced by chemotherapeutic drugs or tumor-related pain [14,15,16,17,18,19,20]. Furthermore, evidence has suggested that kinins and their receptors, kinin B_2_ (B_2_R) and B_1_ (B_1_R) receptors, also play a critical role in peripheral neuropathy induced by chemotherapy and in pain caused by some cancers [21,22,23,24].

Notably, both kinin receptors are co-expressed with the TRPA1 channel on sensory neurons [25,26,27,28,29]. Evidence, both in vitro and in vivo, suggests that the pro-algesic effects of kinins can be mediated, at least partly, by intracellular sensitization of TRPA1 [30,31,32,33]. Indeed, signaling pathways downstream from B_2_R and B_1_R activation, including the PLC/PKC and PKA pathways, are known sensitizers of TRPA1 [30,34]. Thus, these intracellular mechanisms are part of a multifactorial complex that might control the activation of the TRPA1 channel.

Given the above, it is plausible to suggest that kinin receptors and TRPA1 may act together to sustain pain symptoms induced by AIs. However, until now, no study has demonstrated the signaling pathways downstream from B_2_R and B_1_R activation and their interaction with TRPA1 in AIs-induced pain, such as anastrozole. Herein, we present evidence of the crosstalk between the kinins B_2_R and B_1_R and the TRPA1 channel and its contribution to anastrozole-related pain in mice.

## 2. Materials and Methods

### 2.1. Drugs and Reagents

All reagents were of analytical grade and, when not mentioned otherwise, were purchased from Sigma-Aldrich Chemical Company (St. Louis, MO, USA). Anastrozole was acquired from Sun Farmacêutica do Brasil Ltda. (Brasil) and dissolved in 0.5% carboxy methylcellulose (CMC). Bradykinin (Bk; B_2_R agonist), des-Arg^9^-bradykinin (DABk; B_1_R agonist), Icatibant (Icat; antagonist for B_2_R), and des-Arg^9^-[Leu^8^]-bradykinin (DALBk; antagonist for B_1_R) were prepared as stock solutions in phosphate-buffered saline (PBS; 10 mM). Allyl isothiocyanate (AITC, TRPA1 agonist) was dissolved in 10% DMSO. A967079 (A96, TRPA1 antagonist) was dissolved in 10% DMSO and 5% Tween 80 (final concentration administered). The stock solutions of U73122 (U731, a PLC inhibitor) and GF109203X (GF109, a PKC inhibitor) were prepared in 10% absolute ethanol, and the H89 solution (PKA inhibitor) was prepared in 1% DMSO. All the stock solutions were diluted to the desired concentration just before use. The final concentration of stock solutions containing ethanol or DMSO did not exceed 0.5% or 1%, respectively, and did not cause any detectable effect per se. Isotonic solution (0.9% NaCl) was used as vehicle to dilute reagents administered by the oral or intraperitoneal routes, while PBS (10 mM) was used to dilute reagents administered by the intraplantar route. All control groups (vehicles) received the vehicles in which the treatments were soluble. Oral and intraperitoneal treatments were administered in mice at a volume of 10 mL/kg, while intraplantar treatments did not exceed the volume of 20 µL per paw. The doses of the drugs used in this study were based on previous studies [18,33,35,36,37,38].

### 2.2. Animals

Male C57BL/6 mice (25–30 g) were used and maintained temperature-controlled (22 ± 1 °C) under a 12-h light/dark cycle and with standard laboratory chow and water ad libitum. The animals were habituated to the experimental room at least 1 h before the experiments. Experimental protocols followed ethical guidelines established for investigations of experimental pain in conscious animals [39]. The experiments also were performed following the national and international legislation (guidelines of the Brazilian Council of Animal Experimentation Control–CONCEA–and of the U.S. Public Health Service’s Policy on Humane Care and Use of Laboratory Animals–PHS Policy). The experimental protocol was approved by the Institutional Animal Care and Use Committee of the Federal University of Santa Maria (CEUA, process numbers 2304280220/2020 and 3026220520/2020).

Once AIs caused incapacitating pain symptoms, an animal model was used. Using an intact organism is important to obtain an adequate response from these experimental models. In this context, male C57BL/6 mice were used based on their low oestrogen concentrations and previous studies involving AIs-associated pain [13,40]. The number of animals and intensities of noxious stimuli used were the minimum necessary to demonstrate the consistent effects of the treatments. The group size used for each experiment was based on studies with protocols similar to ours [37,38,41], which were confirmed by power calculations (G*Power version 3.1.9.7).

Allocation concealment was performed using a randomization procedure [http://www.randomizer.org/ (accessed on 12 December 2021)] and according to the baseline thresholds before and after the treatment administrations. All experiments were also performed by experimenters blinded to the drug administration or the group to be tested.

### 2.3. Anastrozole-Induced Pain Model

The dose of anastrozole used to induce painful behaviors in mice was based on the human dose of 1 mg recommended by the Food and Drug Administration to treat breast cancer in postmenopausal women [4]. Thus, from the conversion factor from human to mouse indicated by the National Institute of Health [42] and previous studies [43,44], we have used the dose of 0.2 mg/kg to induce a pain model in mice. The experimental protocol consisted of administering anastrozole (0.2 mg/kg) by oral route (p.o.) or vehicle (control group, 10 mL/kg, p.o.; 0.5% CMC). The animals were subjected to behavior assessments after acute treatment (a single anastrozole administration) or prolonged treatment (anastrozole administration for 15 consecutive days). Treatments for pain are usually therapeutic; that is, they are administered after the onset of pain symptoms. Thus, all the experimental protocols were conducted according to this schedule: we first induced the painful symptoms with anastrozole administration, and then we performed antagonist or inhibitor treatments.

### 2.4. Study Design for Behavioral Assessment

Mechanical allodynia (von Frey test) and muscle strength (Grip test) were evaluated before (baseline, BL) and after the anastrozole or vehicle administration (time 0). Next, the animals were submitted to several treatment protocols (see more details below), and these behavioral parameters were evaluated several times (h) after treatments. In most behavioral protocols, overt nociceptive behavior was also evaluated.

#### 2.4.1. Pain Induction by Anastrozole

**Figure 1:**
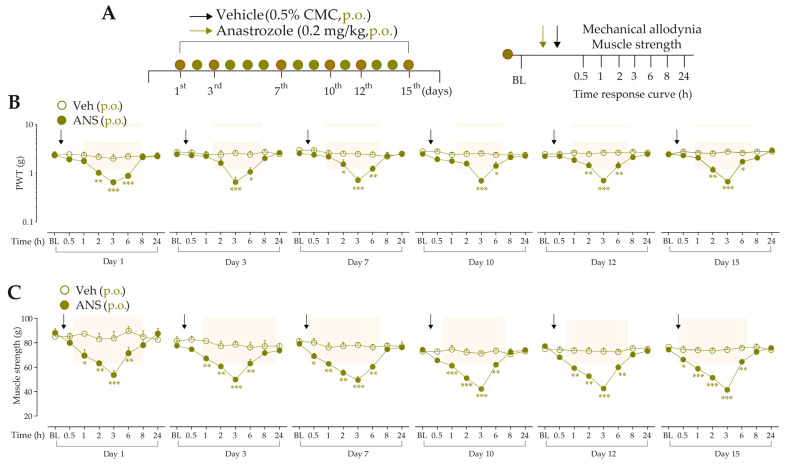
Repeated treatment with anastrozole induces prolonged nociceptive behaviors in mice (**A**) Male C57BL/6 mice were treated by the oral route (p.o.) with anastrozole (0.2 mg/kg) or its vehicle (0.5% CMC) once daily for fifteen days to mimic a 1-year time in humans. On the 1st, 3rd, 7th, 10th, 12th, and 15th days after treatments, a time curve (from 0.5 h up to 24 h) was performed to evaluate mechanical allodynia development and muscle strength loss (**B**,**C**). Baseline (BL) values were measured before anastrozole or vehicle administration. * *p* < 0.05, ** *p* < 0.01, and *** *p* < 0.001 vs. vehicle group. Data were expressed as the mean + SEM (n = 6/group) and analyzed by a two-way ANOVA followed by the Bonferroni post hoc test. The arrows indicate the administration of the anastrozole or vehicle. Veh: vehicle oral administration; ANS: anastrozole oral administration. The arrows indicate the administration of the anastrozole or vehicle.

We investigated whether anastrozole systemic treatment evokes mechanical allodynia and muscle strength loss on a repeated treatment protocol. For this, after evaluation of the baseline paw withdrawal threshold (PWT) and muscle strength of the animals, they were randomized into treatment groups. Mice received anastrozole (0.2 mg/kg, p.o.) or its vehicle (0.5% CMC, p.o.) once a day for 15 consecutive days, which corresponds to a 1-year time in humans [13]. The development of mechanical allodynia and muscle strength loss was evaluated at days 1, 3, 7, 10, 12, and 15 from 0.5 h up to 24 h after each treatment day (following the protocol described in items “2.5.2 Mechanical threshold” and “2.5.3 Muscle strength”). The experimental design is represented in Figure 1A.

#### 2.4.2. Assessment of B_2_R, B_1_R, and TRPA1 Involvement in Anastrozole-Induced Pain

To confirm the contribution of B_2_R, B_1_R, and TRPA1 channels on mechanical allodynia and muscle strength loss induced by anastrozole, the mice received a single administration of Icatibant (100 nmol/kg, intraperitoneal, i.p., B_2_R antagonist) or DALBk (150 nmol/kg, i.p., B_1_R antagonist) or A967079 (100 mg/kg, p.o., TRPA1 channel antagonist) or their vehicles (10 mL/kg, i.p. or p.o.) at 3 h after anastrozole administration. After treatments, the PWT and muscle strength were evaluated at different time points (from 0.5 h to 5 h). The experimental design is represented in Supplementary Figure S1.

**Figure 2:**
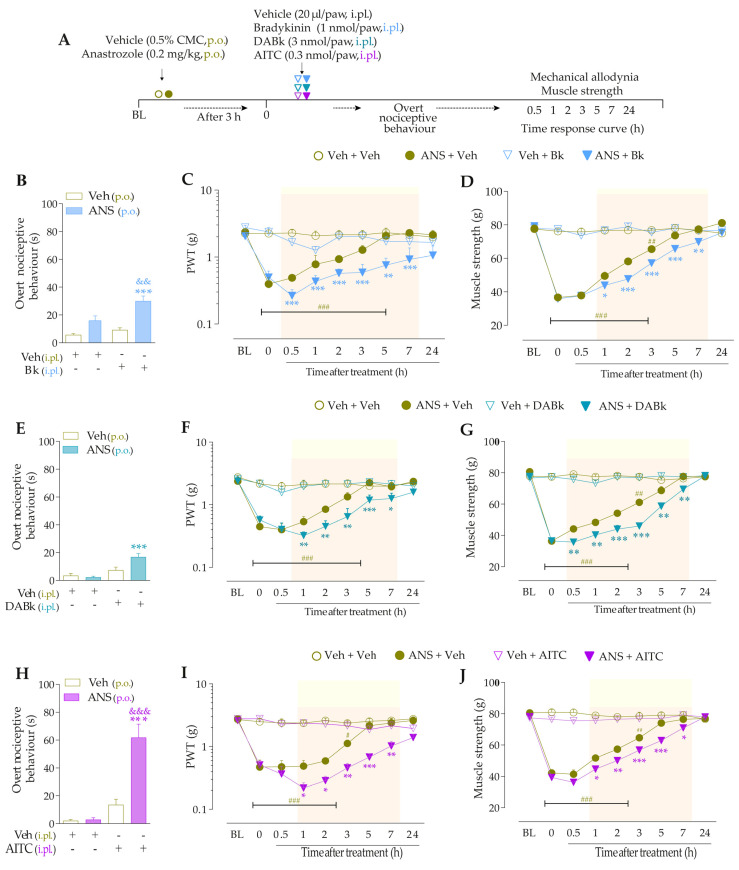
The nociceptive behaviors induced by anastrozole are enhanced and prolonged by local administration of B_2_R, B_1_R, and TRPA1 agonists in mice. (**A**) Male C57BL/6 mice treated by oral route (p.o.) with anastrozole (0.2 mg/kg) presented mechanical allodynia and muscle strength loss compared to their vehicle (0.5% CMC). At 3 h after anastrozole administration (Time 0), the vehicle (20 μL/paw, intraplantar, i.pl.) or sub-nociceptive doses of Bk (1 nmol/paw, i.pl., B_1_R agonist), DABk (3 nmol/paw, i.pl., B_2_R agonist), or AITC (0.3 nmol/paw, i.pl., TRPA1 agonist) were injected by the intraplantar route. The overt nociceptive behavior was immediately evaluated for 10 min (**B**,**E**,**H**); the PWT (**C**,**F**,**I**) and muscle strength (**D**,**G**,**J**) were assessed from 0.5 h up to 24 h after agonists’ sub-nociceptive doses. Baseline (BL) values were measured before the anastrozole or vehicle administration. # *p* < 0.05, ## *p* < 0.01, and ### *p* < 0.001 vs. Veh plus Veh group. * *p* < 0.05, ** *p* < 0.01, and *** *p* < 0.001 vs. ANS plus Veh group. && *p* < 0.01 and &&& *p* < 0.001 vs. Veh plus Bk/AITC group. Data were expressed as the mean + SEM (n = 6/group) and analyzed by one- or two-way ANOVA followed by the Bonferroni post hoc test. Veh: intraplantar, intraperitoneal, or oral vehicle injections; ANS: anastrozole oral administration; Bk: bradykinin intraplantar treatment; PWT: paw withdrawal threshold.

For the first time, it was investigated whether low doses of B_2_R, B_1_R, and TRPA1 channel agonists could enhance painful behaviors in anastrozole-treated mice. For this, the animals were previously treated with anastrozole or vehicles, and after 3 h, they received intraplantar (i.pl.) injections of Bk (1 nmol/paw, a B_2_R agonist), DABk (3 nmol/paw, a B_1_R agonist), or AITC (0.3 nmol/paw, a TRPA1 agonist), all in sub-nociceptive doses, or their vehicles (20 µL/paw, i.pl.). The overt nociceptive behavior was immediately evaluated following the protocol described in item “2.5.1 Overt nociceptive behavior”. PWT and muscle strength were evaluated from 0.5 to 24 h after the stimuli. The experimental design is represented in Figure 2A.

**Figure 3:**
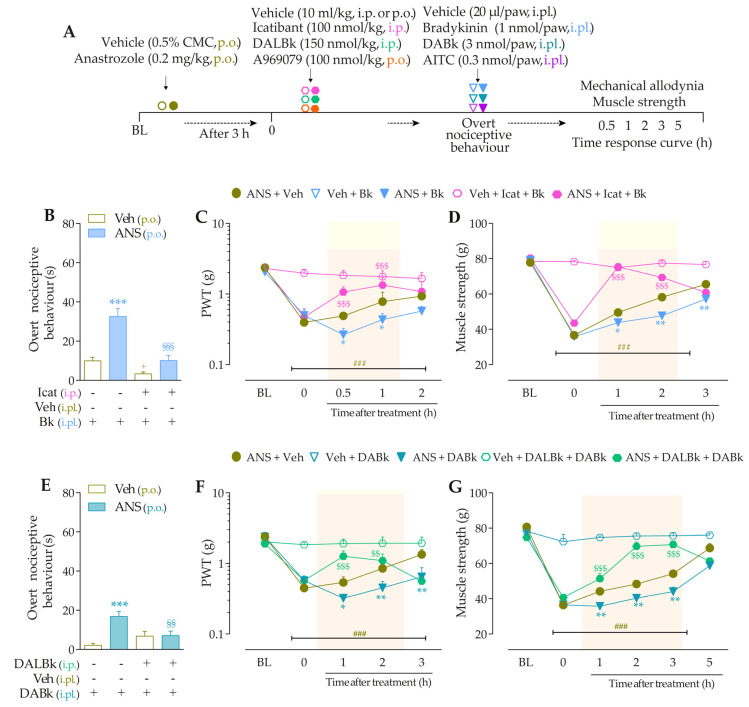

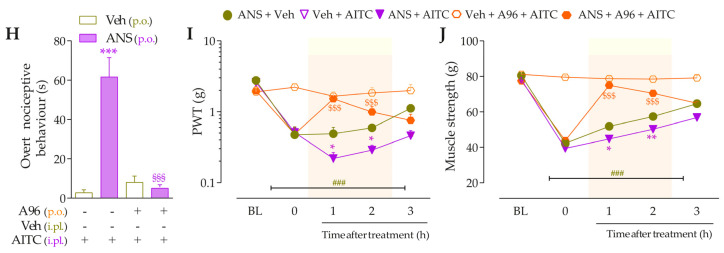
The development of nociceptive behaviors induced by anastrozole is dependent on B_2_ and B_1_ receptors and the TRPA1 channel in mice. (**A**) Male C57BL/6 mice were treated by the oral route (p.o.) with anastrozole (0.2 mg/kg) or its vehicle (0.5% CMC) to induce painful behaviors. At 3 h after anastrozole administration (Time 0), the animals received a single administration of vehicle (10 mL/kg, intraperitoneal, i.p., or p.o.), Icatibant (100 nmol/kg, i.p.), DALBk (150 nmol/kg, i.p.), or A967079 (100 mg/kg, p.o.). After 0.5 h, vehicle (20 μL/paw, intraplantar, i.pl.) or sub-nociceptive doses of their respective agonists, Bk (1 nmol/paw, i.pl.), DABk (3 nmol/paw, i.pl.), or AITC (0.3 nmol/paw, i.pl.), were injected via the intraplantar route. The overt nociceptive behavior was immediately evaluated for 10 min (**B**,**E**,**H**); the PWT (**C**,**F**,**I**) and muscle strength (**D**,**G**,**J**) were assessed from 0.5 h up to 5 h after agonists’ sub-nociceptive doses or until treatments with the antagonists showed an effect. Baseline (BL) values were measured before the anastrozole or vehicle administration. ### *p* < 0.001 vs. Veh plus Veh group. * *p* < 0.05, ** *p* < 0.01, and *** *p* < 0.001 vs. ANS plus Veh group. $$ *p* < 0.01, and $$$ *p* < 0.001 vs. ANS plus Bk/DABk/AITC group. + *p* < 0.05 vs. Veh plus Icat plus Bk group. §§ *p* < 0.01, and §§§ *p* < 0.001. Data were expressed as the mean + SEM (n = 6/group) and analyzed by one- or two-way ANOVA followed by the Bonferroni post hoc test. Veh: intraplantar, intraperitoneal, or oral vehicle injections; ANS: anastrozole oral administration; Bk: bradykinin intraplantar treatment; Icat: Icatibant intraperitoneal treatment; A96: A967079 oral treatment; PWT: paw withdrawal threshold.

To validate the involvement of B_2_R, B_1_R, and TRPA1 channels on the behavioral parameters evaluated above, other animal groups received Icatibant (100 nmol/kg, intraperitoneal, i.p., B_2_R antagonist), DALBk (150 nmol/kg, i.p., B_1_R antagonist), or A967079 (100 mg/kg, p.o., TRPA1 channel antagonist) at 3 h after anastrozole. After 0.5 h, the same animals were treated with sub-nociceptive doses of their respective agonists, Bk, DABk, or AITC, by the intraplantar route, and the overt nociceptive behavior was immediately evaluated for 10 min. In sequence, PWT and muscle strength were assessed until treatments with the antagonists showed an effect. The experimental design is represented in Figure 3A.

#### 2.4.3. Interaction Assessment of the B_2_R, B_1_R, and TRPA1 in Anastrozole-Induced Painful Behaviors

**Figure 4:**
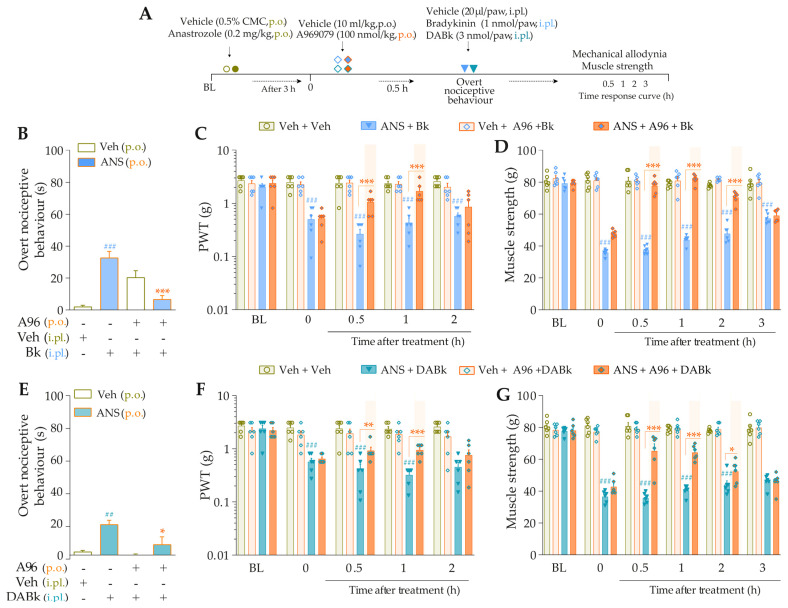
TRPA1 channel contributes to the nociceptive behaviors induced by B_2_R and B_1_R agonists in anastrozole-treated mice. (**A**) Male C57BL/6 mice were treated by the oral route (p.o.) with anastrozole (0.2 mg/kg) or its vehicle (0.5% CMC) to induce painful behaviors. At 3 h after anastrozole administration (Time 0), the animals received a single administration of vehicle (10 mL/kg, p.o.) or A967079 (100 mg/kg, p.o.). After 0.5 h, vehicle (20 μL/paw, intraplantar, i.pl.) or sub-nociceptive doses of agonists, Bk (1 nmol/paw, i.pl.) or DABk (3 nmol/paw, i.pl.), were injected by the intraplantar route. The overt nociceptive behavior was immediately evaluated for 10 min (**B**,**E**); the PWT (**C**,**F**) and strength muscle (**D**,**G**) were assessed until treatments with the antagonist showed an effect. Baseline (BL) values were measured before the anastrozole or vehicle administration. ## *p* < 0.01 and ### *p* < 0.001 vs. Veh plus Veh group. * *p* < 0.05, ** *p* < 0.01, and *** *p* < 0.001 vs. ANS plus Bk/DABk group. Data were expressed as the mean + SEM (n = 6/group) and analyzed by one- or two-way ANOVA followed by the Bonferroni post hoc test. Veh: intraplantar, intraperitoneal, or oral vehicle injections; ANS: oral administration of anastrozole; Bk: bradykinin intraplantar treatment; A96: A967079 oral treatment; PWT: paw withdrawal threshold.

**Figure 5:**
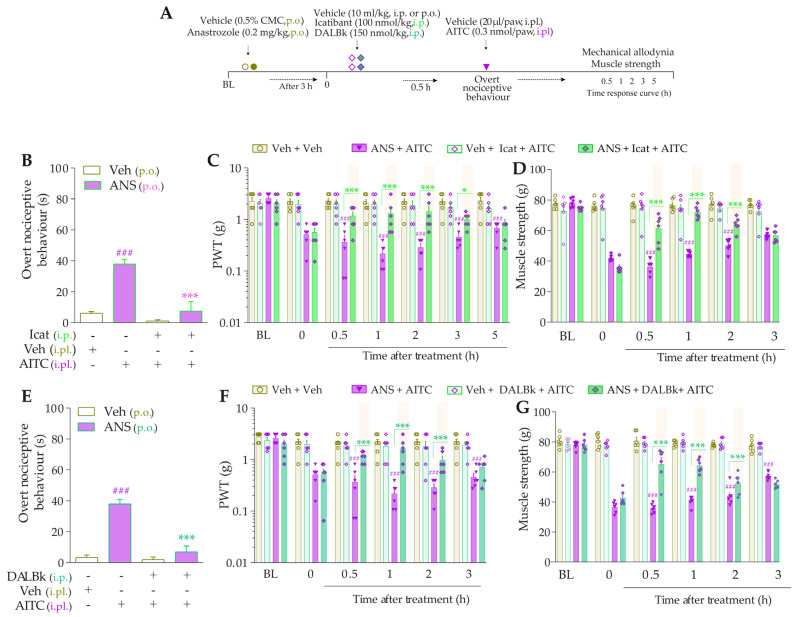
Kinin B_2_ and B_1_ receptors contribute to the nociceptive behaviors induced by the TRPA1 agonist in anastrozole-treated mice. (**A**) Male C57BL/6 mice were treated by the oral route (p.o.) with anastrozole (0.2 mg/kg) or its vehicle (0.5% CMC) to induce painful behaviors. At 3 h after anastrozole administration (Time 0), the animals received a single administration of Icatibant (150 nmol/kg, i.p.) or DALBk (100 nmol/kg, i.p.). After 0.5 h, vehicle (20 μL/paw, intraplantar, i.pl.) or AITC (0.3 nmol/paw, i.pl.; sub-nociceptive dose) were injected by the intraplantar route. The overt nociceptive behavior was immediately evaluated for 10 min (**B**,**E**); the PWT (**C**,**F**) and strength muscle (**D**,**G**) were assessed until treatments with the antagonist showed an effect. Baseline (BL) values were measured before the anastrozole or vehicle administration. ### *p* < 0.001 vs. Veh plus Veh group. * *p* < 0.05, *** *p* < 0.001 vs. ANS plus AITC group. Data were expressed as the mean + SEM (n = 6/group) and analyzed by one- or two-way ANOVA followed by the Bonferroni post hoc test. Veh: intraplantar, intraperitoneal, or oral vehicle injections; ANS: oral administration of anastrozole; Icat: Icatibant intraperitoneal treatment; PWT: paw withdrawal threshold.

We explored whether the interaction between both kinin receptors and TRPA1 might be essential to mediate the anastrozole-induced pain. The animals were previously treated with anastrozole or vehicle, and after 3 h, they received Icatibant, DALBk, or A967079. After 0.5 h, the B_2_R (Icatibant) or B_1_R (DALBk) antagonist-treated animals received the intraplantar injection of the TRPA1 agonist, AITC (0.3 nmol/paw, i.pl.). The animal groups treated with the systemic TRPA1 (A967076) antagonist received the intraplantar injection of either B_2_R or B_1_R agonists (Bk or DABK, 1 or 3 nmol/paw, respectively). The overt nociceptive behavior was evaluated immediately after intraplantar agonist administration for 10 min. In sequence, PWT and muscle strength were assessed until treatments with the antagonists showed an effect. The experimental designs are represented in Figures 4A and 5A.

**Figure 6:**
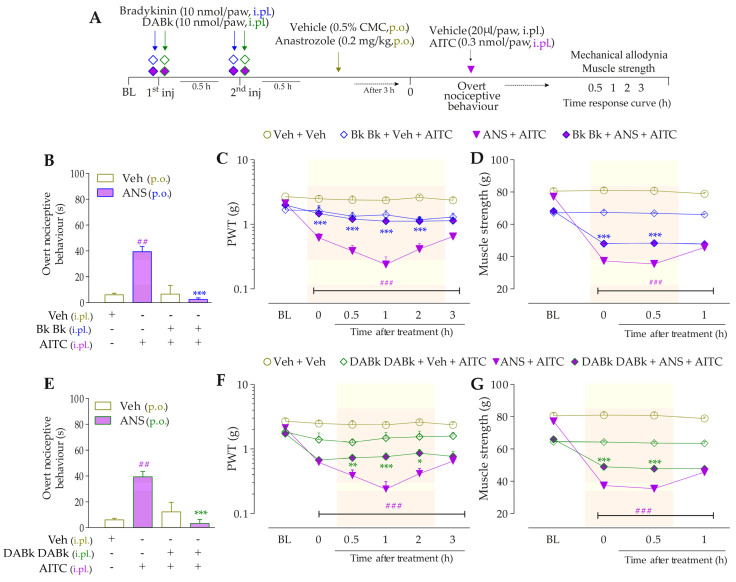
Kinin B_2_ and B_1_ receptors are required to stimulate TRPA1 and cause nociceptive behaviors in anastrozole-treated mice. (**A**) Male C57BL/6 mice received two repeated intraplantar injections of Bk (10 nmol/paw, intraplantar, i.pl.) or DABk (10 nmol/paw, i.pl.) with 0.5 h of the interval between the injections. After 0.5 h of the last administration, the animals were treated by the oral route (p.o.) with anastrozole (0.2 mg/kg) or its vehicle (0.5% CMC) to induce painful behaviors. After 3 h, the animals received a single injection of vehicle (20 μL/paw, i.pl.) or AITC (0.3 nmol/paw, i.pl.; sub-nociceptive dose). The overt nociceptive behavior was immediately evaluated for 10 min (**B**,**E**); the PWT (**C**,**F**) and strength muscle (**D**,**G**) were assessed until the desensitization protocol showed an effect. Baseline (BL) values were measured before the anastrozole or vehicle administration. ## *p* < 0.01 and ### *p* < 0.001 vs. Veh plus Veh group. * *p* < 0.05, ** *p* < 0.01, and *** *p* < 0.001 vs. ANS plus AITC group. Data were expressed as the mean + SEM (n = 6/group) and analyzed by one- or two-way ANOVA followed by the Bonferroni post hoc test. Veh: intraplantar or oral vehicle injections; ANS: oral administration of anastrozole; Bk: bradykinin intraplantar treatment; PWT: paw withdrawal threshold.

To confirm the results about the interaction between B_2_R and B_1_R and TRPA1 in the pain symptoms induced by anastrozole, we use an in vivo desensitization protocol (following the protocol described in item “2.5.4 Desensitization protocol”). After 0.5 h of desensitization protocol, the animals received anastrozole or vehicle, and after 3 h, the desensitized animals received an intraplantar injection of AITC (0.3 nmol/paw, i.pl.), and the overt nociceptive behavior was immediately evaluated. After that, PWT and muscle strength were assessed several times (h). The experimental design is represented in Figure 6A.

#### 2.4.4. Intracellular Pathways Dependent on Kinin Receptor Activation and TRPA1 Sensitization

**Figure 7:**
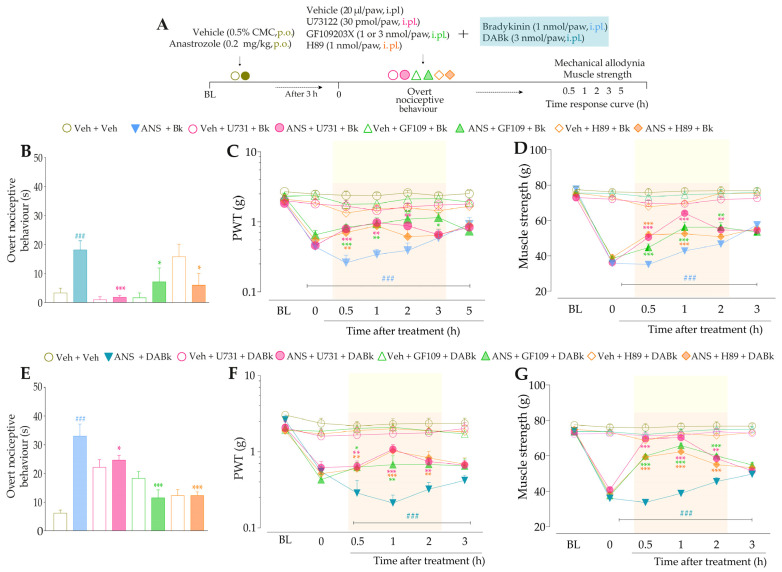
The signaling pathways downstream from the kinin B_2_ and B_1_ receptors are activated and contribute to the nociceptive behaviors induced by kinin agonists in anastrozole-treated mice. (**A**) Male C57BL/6 mice were treated by the oral route (p.o.) with anastrozole (0.2 mg/kg) or its vehicle (0.5% CMC) to induce painful behaviors. At 3 h after anastrozole administration (Time 0), the animals received the intraplantar (i.pl.) co-injection of U73122 (30 pmol/paw, i.pl., PLC inhibitor), GF109203X (1 or 3 nmol/paw, i.pl., PKC inhibitor), or H89 (1 nmol/paw, i.pl., PKA inhibitor), and a sub-nociceptive dose of Bk (1 nmol/paw, i.pl.) or DABk (3 nmol/paw, i.pl.). The overt nociceptive behavior was immediately evaluated for 10 min (**B**,**E**); the PWT (**C**,**F**) and strength muscle (**D**,**G**) were assessed until treatments with the inhibitors showed an effect. Baseline (BL) values were measured before the anastrozole or vehicle administration. ### *p* < 0.001 vs. Veh plus Veh group. * *p* < 0.05, ** *p* < 0.01, and *** *p* < 0.001 vs. ANS plus Bk group. Data were expressed as the mean + SEM (n = 6/group) and analyzed by one- or two-way ANOVA followed by the Bonferroni post hoc test, except for the U73122 effect on overt nociceptive behavior induced by ANS plus Bk and the H89 effect on overt nociceptive behavior induced by ANS plus DABk, which were analyzed by Student’s *t*-test. Veh: intraplantar or oral vehicle injections; ANS: oral administration of anastrozole to induce the pain model; Bk: bradykinin intraplantar treatment; U731: U73122, a PLC inhibitor; GF109: GF109203X, a PKC inhibitor; PWT: paw withdrawal threshold.

We also examined the participation of both intracellular signaling pathways, mediated by PLC/PKC or PKA, in the anastrozole-induced pain. For this, animals previously treated (3 h before) with anastrozole or vehicle received intraplantar co-injection containing either an inhibitor of PLC (U73122, 30 pmol/paw, i.pl.), PKC (GF109203X, 1 or 3 nmol/paw, i.pl.) or PKA (H89, 1 nmol/paw, i.pl.) plus Bk (1 nmol/paw, i.pl.) or DABK (3 nmol/paw, i.pl.). Immediately after intraplantar injection of agonists, overt nociceptive behavior was evaluated. Next, PWT and muscle strength were assessed until treatments with the inhibitors showed an effect. The experimental design is represented in Figure 7A.

**Figure 8:**
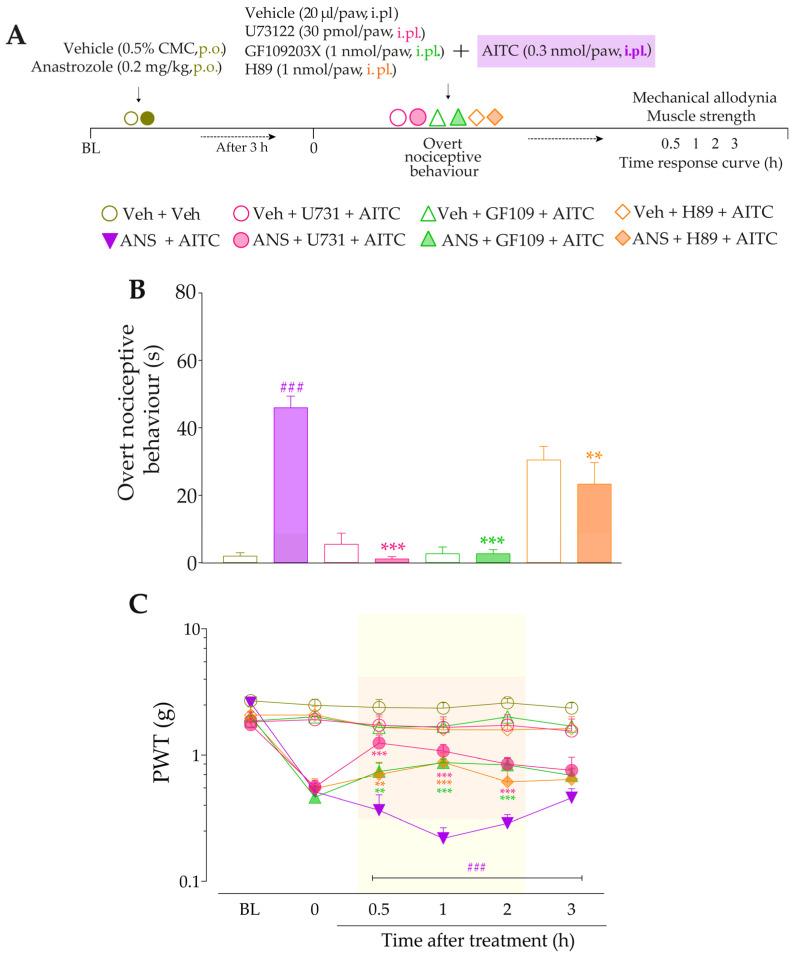

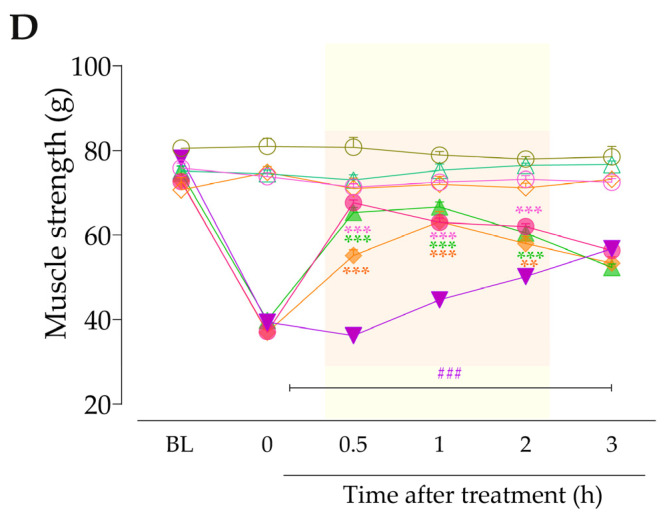
PLC/PKC and PKA signaling pathway inhibition attenuated the nociceptive behaviors induced by the TRPA1 agonist in anastrozole-treated mice. (**A**) Male C57BL/6 mice were treated by the oral route (p.o.) with anastrozole (0.2 mg/kg) or its vehicle (0.5% CMC) to induce painful behaviors. At 3 h after anastrozole administration (Time 0), the animals received the intraplantar (i.pl.) co-injection of U73122 (30 pmol/paw, i.pl., PLC inhibitor), GF109203X (1 nmol/paw, i.pl., PKC inhibitor), or H89 (1 nmol/paw, i.pl., PKA inhibitor), and a sub-nociceptive dose of AITC (0.3 nmol/paw, i.pl.). The overt nociceptive behavior was immediately evaluated for 10 min (**B**); the PWT (**C**) and strength muscle (**D**) were assessed until treatments with the inhibitors showed an effect. Baseline (BL) values were measured before the anastrozole or vehicle administration. ### *p* < 0.001 vs. Veh plus Veh group. ** *p* < 0.01, and *** *p* < 0.001 vs. ANS plus AITC group. Data were expressed as the mean + SEM (n = 6/group) and analyzed by one- or two-way ANOVA followed by the Bonferroni post hoc test. Veh: intraplantar or oral vehicle injections; ANS: oral administration of anastrozole; U731: U73122, a PLC inhibitor; GF109: GF109203X, a PKC inhibitor; PWT: paw withdrawal threshold.

Next, we evaluated whether signaling pathways downstream from B_2_R and B_1_R activation could be contributing to TRPA1 sensitization in this model. At 3 h after treatments with anastrozole or vehicle, the animals received intraplantar co-injection containing either inhibitors of PLC, PKC, or PKA plus AITC (0.3 nmol/paw, i.pl.), and the overt nociceptive behavior was immediately evaluated. Next, PWT and muscle strength were assessed until treatments with the inhibitors showed an effect. The experimental design is represented in Figure 8A.

### 2.5. Behavioral Experiments

#### 2.5.1. Overt Nociceptive Behavior

We evaluated the capacities of B_2_R (Bk), B_1_R (DABk), and TRPA1 (AITC) channel agonists in increasing the nociceptive response induced by anastrozole (all at sub-nociceptive doses). For this, the animals were individually placed in chambers (transparent glass cylinders of 20 cm diameter) and adapted for 10 min. Immediately after the intraplantar injection of agonists in the right hind paw, the animals were observed for 10 min. The amount of time spent (in seconds, s) licking and lifting the injected paw was considered indicative of overt nociceptive behavior [13,44].

#### 2.5.2. Mechanical Allodynia

The assessment of mechanical allodynia was carried out using flexible nylon filaments (von Frey) of increasing stiffness (0.02–10 g) by the up-and-down method [45,46]. The von Frey test is widely used in preclinical and clinical settings for measuring pain [46,47]. The PWT was calculated from the resulting scores, as previously described [48]. The PWT was expressed in grams (g), and a significant decrease in the PWT compared with the baseline values was considered as mechanical allodynia.

#### 2.5.3. Muscle Strength

The muscle strength test in mice was evaluated using a grip strength meter (Grip Strength meter EFF 305–IInsight, Ribeirão Preto, SP, Brazil) [49], a test used in preclinical and clinical settings for the study of musculoskeletal pain [50,51]. The test consists of the experimenter gently holding the mouse by the base of the tail, allowing the animal to grab the metal bar with the forelimbs and hindlimbs before being gently pulled until it releases its grip. The forelimbs and hindlimbs muscle strength, expressed in grams (g), was measured three times per mouse with at least 1 min between measurements.

#### 2.5.4. Desensitization Protocol

Although the presence of B_2_R on nociceptive primary afferent neurons is well established, evidence also demonstrates that B_1_R is expressed in some small and medium diameter neurons and peptidergic and non-peptidergic C fibers [26,29,52,53]. In this sense, the desensitization protocol was performed on both kinin B_2_R and B_1_R, based on and adapted from Ferreira et al. [35]. The mice received two repeated intraplantar injections of Bk (10 nmol/paw, i.pl.) or DABK (10 nmol/paw, i.pl.) with a 0.5 h interval between the injections.

### 2.6. Statistical Analysis

The results were expressed as the mean + Standard Error of the Mean (SEM), which were expressed as geometric means accompanied by their respective 95% confidence limits. The Kolmogorov-Smirnov test was used to assess the normality of the data. To meet parametric assumptions, data of mechanical threshold were log-transformed before analyses. Parametric data were analyzed by Student’s *t*-test or one- or two-way ANOVA followed by Bonferroni’s post hoc test, using the GraphPad Prism 8.0 software (San Diego, CA, USA). Post hoc tests were performed only when the F-value achieved the necessary level of statistical significance (*p* < 0.05) and when there was no significant variance in homogeneity [54]. The percentages of maximum inhibition (Imax) were calculated for the maximally developed responses compared to baseline values or the control group.

## 3. Results

### 3.1. Systemic Anastrozole Induces Prolonged Pain Symptoms in Mice

Mice daily treated with anastrozole developed mechanical allodynia (from 2 h up to 6 h) (Figure 1B) and presented reduced muscle strength (from 0.5 h up to 6 h) (Figure 1C) at days 1, 3, 7, 10, 12, and 15 after anastrozole treatment when compared to the vehicle group. The development of maximum mechanical allodynia and muscle strength loss occurred 3 h after oral anastrozole treatment, returning to basal levels 8 h after anastrozole. Based on this, the time of 3 h on the first day after anastrozole administration was chosen for subsequent experiments.

### 3.2. Kinin B_2_ and B_1_ Receptors and the TRPA1 Channel Contribute to Pain Induced by Anastrozole

Corroborating other studies [13,44], Icatibant, DALBk, or A967076 reduced the mechanical allodynia and muscle strength loss in anastrozole-treated animals (Supplementary Figure S1). Icatibant (100 nmol/kg, i.p.) (Supplementary Figure S1B) or DALBk (150 nmol/kg, i.p.) (Supplementary Figure S1D) reduced the anastrozole-induced mechanical allodynia from 0.5 up to 2 h after their administration, with inhibitions of 56 ± 7% and 100% at 1 h after their treatments, respectively. A967079 (100 mg/kg, p.o.) (Supplementary Figure S1F) reduced the mechanical allodynia induced by anastrozole from 0.5 up to 1 h after its administration, with inhibitions of 47 ± 10% at 1 h. Icatibant (Supplementary Figure S1C), DALBk (Supplementary Figure S1E), or A967079 (Supplementary Figure S1G) also reduced the muscle strength loss induced by anastrozole from 0.5 up to 3 h after their administration, with inhibitions of 89 ± 6%, 87 ± 11%, and 92 ± 5% at 1 h, respectively.

The intraplantar (i.pl.) injection of Bk (1 nmol/paw) (Figure 2B), DABk (3 nmol/paw) (Figure 2E), or AITC (0.3 nmol/paw) (Figure 2H), all in sub-nociceptive doses, increased paw licking and lifting time in anastrozole-treated compared to vehicle-treated animals, an indication of overt nociceptive behavior. Anastrozole (0.2 mg/kg, p.o.) induced mechanical allodynia (Time 0, Figure 2C,F,I) and muscle strength loss (Time 0, Figure 2D,G,J) in mice at 3 h after its administration when compared to BL values. Intraplantar Bk, DABk, or AITC enhanced and prolonged the mechanical allodynia (Figure 2C,F,I) and muscle strength loss (Figure 2D,G,J) from 0.5 up to 7 h after its injection in anastrozole-treated animals when compared to the anastrozole plus vehicle group.

Icatibant (100 nmol/kg, i.p., 0.5 h prior) (Figure 3B), DALBk (150 nmol/kg, i.p., 0.5 h prior) (Figure 3E), or A967079 (100 mg/kg, p.o., 0.5 h prior) (Figure 3H) markedly prevented the Bk-, DABk-, or AITC- induced overt nociceptive behavior in anastrozole-treated mice with inhibition of 68 ± 7%, 65 ± 11%, and 91 ± 3%, respectively. Icatibant reduced the mechanical allodynia (from 0.5 h up to 1 h) (Figure 3C) and muscle strength loss (from 1 h up to 2 h) (Figure 3D) with inhibitions of 55 ± 23% (at 1 h) and 89 ± 2% (at 1 h), respectively, after its administration. DALBk reduced the mechanical allodynia (Figure 3F) and muscle strength loss (Figure 3G) from 1 h up to 2 h (inhibition of 52 ± 13% at 1 h) and from 1 h up to 3 h (inhibition of 79 ± 6% at 2 h), respectively, after its administration. A967079 reduced the mechanical allodynia (Figure 3I) and muscle strength loss (Figure 3J) from 1 h up to 2 h (inhibition of 54 ± 7% and 90 ± 7% at 1 h, respectively) after its administration. As expected, sub-nociceptive doses of the Bk, DABk, or AITC agonists did not induce overt nociceptive behavior, mechanical allodynia, or muscle strength loss in animals previously treated with vehicle (Figure 3).

### 3.3. Interaction of Kinin B_2_ and B_1_ Receptors and the TRPA1 Channel Sustain the Anastrozole-Induced Pain

Intraplantar Bk (1 nmol/paw, i.pl.) and DABk (3 nmol/paw, i.pl.), at sub-nociceptive doses, induced significant overt nociceptive behavior (Figure 4B,E) and enhanced the mechanical allodynia (Figure 4C,F) and the muscle strength loss (Figure 4D,G) in anastrozole-treated animals compared to the vehicle plus vehicle group. A967079 (100 mg/kg, p.o., 0.5 h prior) markedly prevented the sensitizing effect induced by Bk (Figure 4B) and DABk (Figure 4E) in anastrozole-treated animals on the overt nociceptive behavior with inhibitions of 79 ± 7% and 66 ± 26%, respectively, compared to the anastrozole plus Bk or DABk groups. A967079 also prevented Bk- and DABk-induced responses on mechanical allodynia from 0.5 h up to 1 h for both agonists (inhibition of 77 ± 20% and 30 ± 5% at 1 h, respectively) (Figure 4C,F) and muscle strength loss from 0.5 h up to 2 h for both agonists (inhibition of 100% and 65 ± 11% at 0.5 h, respectively) (Figure 4D,G).

The sensitizing effect induced by TRPA1 agonist AITC (0.3 nmol/paw, i.pl.) on overt nociceptive behavior, mechanical allodynia, and muscle strength loss was prevented by pre-treatment with B_2_R and B_1_R antagonists. Icatibant (100 nmol/kg, i.p., 0.5 h prior) markedly inhibited the responses induced by AITC (0.3 nmol/paw, i.pl.) on the overt nociceptive behavior (Figure 5B; inhibition of 80 ± 16%), mechanical allodynia (Figure 5C), and muscle strength loss (Figure 5D) from 0.5 h up to 3 h (inhibition of 49 ± 17% at 2 h) and from 0.5 h up to 2 h (inhibition of 90 ± 7% at 1 h) after its administration, respectively. The sensitizing effect induced by AITC in anastrozole-treated animals also was prevented by the pre-treatment with DALBk (150 nmol/kg, i.p., 0.5 h prior) on the overt nociceptive behavior (Figure 5E; inhibition of 81 ± 10%), mechanical allodynia (Figure 5F), and muscle strength loss (Figure 5G) from 0.5 h up to 2 h (inhibition of 62 ± 18% at 1 h and inhibition of 99 ± 1% at 1 h, respectively). These results suggest the cooperation between kinin B_2_R and B_1_R and TRPA1 channel to mediate nociceptive parameters induced by anastrozole.

The desensitization protocol by intraplantar injection of Bk (Figure 6B) or DABk (Figure 6E) completely abrogated the AITC-induced overt nociceptive behaviors in anastrozole-treated mice (inhibitions of 93 ± 3% and 91 ± 7%, respectively) compared to anastrozole plus AITC-treated non-desensitized mice. In these same animals, the local desensitization with Bk and DABk also abolished the mechanical allodynia from 0.5 h up to 3 h (inhibition of 47 ± 10% at 0.5 h and 27 ± 5% at 1 h, respectively) (Figure 6C,F) and muscle strength loss from 0.5 h up to 1 h (inhibition of 33 ± 2% and 32 ± 2% at 0.5 h, respectively) (Figure 6D,G) after its injections, when compared to the non-desensitized group anastrozole plus AITC.

### 3.4. Intracellular Pathways Dependent on Kinin B_2_ and B_1_ Receptor Activation Cooperate to Sensitize TRPA1 in Anastrozole-Treated Mice

Firstly, we evaluated the activation of signaling pathways downstream from the B_2_R in the anastrozole-induced pain model. The local inhibition of PLC (U73122, 30 pmol/paw, i.pl.) or PKC (GF109203X, 1 nmol/paw, i.pl.) significantly attenuated the overt nociceptive behavior induced by a sub-nociceptive dose of Bk in animals treated with anastrozole (Figure 7B), with inhibitions of 25 ± 5% and 65 ± 8%, respectively. Moreover, the U73122 and GF109203X inhibitors reduced Bk-induced responses on mechanical allodynia (inhibition of 42 ± 9% at 1 h and 45 ± 13% at 2 h after its injections, respectively) (Figure 7C) and muscle strength loss (inhibition of 60 ± 3% and 38 ± 8% at 1 h after its injections, respectively) (Figure 7D) in anastrozole-treated mice. In addition, the local inhibition of PKA (H89, 1 nmol/paw, i.pl.) decreased the Bk-induced responses on the overt nociceptive behavior (inhibition of 62 ± 3%) (Figure 7B), mechanical allodynia (inhibition of 36 ± 10% at 1 h after its injection) (Figure 7C), and muscle strength loss (inhibition of 39 ± 3% at 0.5 h after its injection) (Figure 7D) in anastrozole-treated mice.

After that, we assessed the contribution of signaling pathways downstream from the B_1_R. The overt nociceptive behavior induced by a sub-nociceptive dose of DABk in animals treated with anastrozole was attenuated when DABK was co-injected with inhibitors of PLC (U73122) or PKA (H89), with inhibitions of 60 ± 23% and 67 ± 23%, respectively (Figure 7E). Furthermore, both inhibitors also reduced the responses induced by DABk on mechanical allodynia (inhibition of 35 ± 6% and 33 ± 5% at 1 h after its injections, respectively) (Figure 7F) and on muscle strength loss (inhibition of 89 ± 3% and 66 ± 6% at 1 h after its injections, respectively) (Figure 7G) in anastrozole-treated mice. The PKC inhibitor (GF109203X) at a dose of 3 but not 1 nmol/paw attenuated the overt nociceptive behavior (inhibition of 89 ± 4%) (Figure 7E), mechanical allodynia (inhibition of 38 ± 10% at 1 h after its injection) (Figure 7F), and muscle strength loss (inhibition of 76 ± 4% at 1 h after its injection) (Figure 7G) induced by a sub-nociceptive dose of DABk in anastrozole-treated animals.

Next, we evaluated whether signaling pathways downstream from B_2_R and B_1_R activation could be contributing to TRPA1 activation in this model. The inhibition of PLC and PKC by U73122 and GF109203X, respectively, reduced the overt nociceptive behavior induced by a sub-nociceptive dose of AITC in animals treated with anastrozole compared to the anastrozole plus AITC group (Figure 8B), with inhibitions of 94 ± 3% and 97 ± 2%, respectively. Furthermore, both inhibitors, U73122 and GF109203X, reduced AITC-induced responses on mechanical allodynia (inhibition of 39 ± 9% and 27 ± 2% at 0.5 h after its injections, respectively) (Figure 8C) and muscle strength loss (inhibition of 75 ± 2% at 1 h and 69 ± 3% at 0.5 h after its injections, respectively) in previously anastrozole-treated mice (Figure 8D). Moreover, the inhibition of PKA by H89 attenuated the AITC-induced responses on overt nociceptive behavior (inhibition of 49 ± 1%) (Figure 8B), mechanical allodynia (inhibition of 28 ± 7% at 1 h after its injection) (Figure 8C), and muscle strength loss (inhibition of 52 ± 7 % at 1 h after its injection) in previously anastrozole-treated mice (Figure 8D).

## 4. Discussion

AIs are the mainstay of endocrine treatment for postmenopausal women diagnosed with hormone receptor-positive breast cancer [3,7]. However, they cause musculoskeletal pain, leading to non-adherence or discontinuation of therapy [8]. Although the participation of the TRPA1 channel and kinins B_1_R and B_2_R in AI-induced pain is well characterized [13,44], the interaction between these receptors in the development and maintenance of pain remains unknown. In this study, single or repeated (for 15 days) anastrozole administration caused pain symptoms (mechanical allodynia and muscle strength loss) in mice. The local administration of B_2_R, B_1_R, or TRPA1 agonists induced overt nociceptive behavior and enhanced and prolonged the pain parameters in anastrozole-treated mice. All painful behaviors mentioned above were attenuated by B_2_R, B_1_R, or TRPA1 antagonists. The interaction between B_2_R and B_1_R and the TRPA1 channel was confirmed using agonists and antagonists of these receptors and an in vivo desensitization protocol. Furthermore, we showed that signaling pathways downstream from B_2_R and B_1_R activation involving PLC, PKC, and PKA are crucial factors in sensitizing TRPA1 and contributing to sustaining anastrozole-induced pain symptoms.

In clinical practice, patients receive anastrozole for long periods, and many develop musculoskeletal pain that persists throughout the treatment [3,8]. In this study, we showed for the first time that anastrozole treatment for 15 days induced pain behavior in mice without causing desensitization, similar to other AIs (exemestane and letrozole) [13]. The maximum pain behavior occurred 3 h after anastrozole treatment, which is consistent with the peak plasma concentration of anastrozole in humans (2–4 h after administration) [55].

Local exposure to agonists of kinin receptors (B_2_R or B_1_R) and TRPA1 (at doses that generally do not cause nociception) is associated with immediate nociceptive responses lasting for a few minutes and more prolonged nociceptive behaviors in animals previously sensitized [13,38,40,56]. Although we have utilized sub-nociceptive doses of B_2_R and B_1_R and TRPA1 agonists, it is worth mentioning that bradykinin (a B_2_R agonist) and AITC (a TRPA1 channel agonist) can evoke pain behaviors in a dose-dependent manner in naive animals [35,57,58]. However, even with increasing DABk doses (a B_1_R agonist), it does not induce painful behaviors in naive animals [23,35,59], only in animals previously sensitized by different pain models [23,57,59], such as after anastrozole treatment. Corroborating with these results, we showed for the first time that the intraplantar administration of B_2_R, B_1_R, or TRPA1 agonists caused overt nociceptive behavior and enhanced the mechanical allodynia and loss of muscle strength of anastrozole-treated but not vehicle-treated mice, which were reduced by pre-treatment with their respective antagonists. In fact, systemic B_2_R, B_1_R, or TRPA1 antagonists also reduced the mechanical allodynia and loss of muscle strength of AIs-treated mice [13,44].

Although AIs activate the TRPA1 channel, the AI concentration needed for its activation in vitro is higher than that in patient plasma [13], suggesting that additional factors interact to increase the sensitivity of TRPA1 to AIs. Indeed, previous studies have reported that TRPA1 endogenous agonists are pro-algesic mediators that contribute to sensitizing TRPA1 and generating AI-induced painful conditions [13,40]. Furthermore, PAR-2 activation also sensitizes TRPA1, causing AIs-induced pain [13]. Therefore, the current findings indicate that other pro-algesic factors or signaling pathways that might activate or sensitize TRPA1 may act along with AIs to trigger pain symptoms. In this sense, B_1_R and B_2_R activation could sensitize the TRPA1 channel once they are co-expressed in sensory neurons and participate in the painful stimulus transmission [25,27,28,29,60]. Once studies have shown that B_2_R and B_1_R interact with TRP channels, including TRPA1 [30,31,35,37,61], we hypothesized that B_2_R and B_1_R activation is a prerequisite for enhancing the sensitivity of TRPA1 to AIs.

Confirming this hypothesis, B_2_R and B_1_R antagonists prevented the sensitizing effect of the TRPA1 agonist on pain parameters in anastrozole-treated animals. Furthermore, the TRPA1 antagonist also prevented pain behaviors induced by B_1_R and B_2_R agonists, similar to other studies [33,62]. Our results are also consistent with previous studies showing that the pharmacological inhibition or genetic deletion of TRPA1 reduces pain responses to intraplantar injections of B_2_R and B_1_R agonists [30,33].

The repeated or continuous stimulation of G protein-coupled receptors (GPCRs) results in a desensitization process, i.e., a loss or reduction of their response [63]. Utilizing an in vivo desensitization protocol, we reinforced the interaction between B_2_R and B_1_R and the TRPA1 channel in the pain symptoms induced by anastrozole. The intraplantar TRPA1 agonist injection in previously anastrozole-treated mice failed to cause pain-related behaviors in animals submitted to desensitization protocol by B_2_R and B_1_R agonists. To date, there have been no studies performing the B_1_R desensitization protocol. However, both B_2_R and B_1_R receptors can be expressed in sensory neurons [29]. Once Ferreira et al. [35] demonstrated that repeated administration of Bk resulted in B_2_R desensitization, a GPCR, we adapted the experimental protocol for B_1_R desensitization. Together, these results suggest that the TRPA1 channel might be sensitized downstream from B_2_R and B_1_R activation, contributing to pain symptoms induced by anastrozole. However, the underlying pathways activated by kinin receptors and the putative sensitizers of TRPA1 involved in this process have not yet been elucidated. In this sense, we investigated the involvement of PLC/PKC- and PKA-dependent signaling pathways on TRPA1 sensitization from B_2_R and B_1_R activation.

B_2_R and B_1_R stimulation leads to the activation of the PLC/PKC-dependent signaling pathway [64], resulting in increased cytosolic calcium (Ca^2+^) levels and diacylglycerol release. Diacylglycerol can activate PKC or be hydrolyzed by diacylglycerol lipase to generate 2-arachidonolyglycerol, followed by arachidonic acid formation dependent on monoacylglycerol lipase [64,65]. The PLC/PKC pathway activation contributes to sensitizing sensory neurons, and therefore, it is critical for developing acute and chronic pain conditions [37,66,67,68]. Once PLC/PKC pathway inhibition reduces the painful behaviors [37,67,68,69], we evaluated the involvement of this signaling pathway dependent on B_2_R and B_1_R activation in anastrozole-treated mice. Our findings indicate that PLC and PKC activation sustains the sensitizing effect of B_2_R and B_1_R agonists in anastrozole-treated animals once inhibition of their activity significantly attenuates the pain symptoms in animals. Consistent with these findings, previously published data has confirmed that activating the PLC/PKC signaling pathways dependent on kinin receptors is essential to assist hypersensitivity in several pain conditions in rodents [31,33,35,37,59,70].

Although less evident, some studies suggest that kinin receptors might sensitize nociceptors dependent on PKA activation [31,35,71]. Indeed, PKA signaling mediates different types of pathological pain [72,73,74,75], while its inhibition reduces the neural response [31,32] and pain behaviors [74,76,77]. Here, we identified that PKA activation seems to be critical in mediating the anastrozole-induced pain because its blockade reversed B_2_R and B_1_R agonist-induced sensitizing effects. It is plausible that kinin receptor activation leads to the activation of PKA by indirect mechanisms dependent on prostaglandin production. Arachidonic acid, formed by PLC/PKC signaling, is rapidly converted by cyclooxygenases to prostaglandins, which prostanoid receptors activate, stimulating cAMP production by adenylyl cyclase. cAMP activates PKA, which phosphorylates signaling proteins, including the TRPA1 channel, contributing to its activation [31,34,78]. Nonetheless, more studies are necessary to elucidate PKA activation followed by B_2_R and B_1_R activation.

The TRPA1 channel might be sensitized by intracellular mechanisms dependent on B_2_R and B_1_R activation [30,31,33,71]. The signaling molecules that activate TRPA1 downstream of PLC/PKC, or PKA are widely debated in the literature [30,32,34]. The substances metabolized during arachidonic acid hydrolysis, the release of Ca^2+^ from intracellular stores, and TRPA1 phosphorylation by PKC and PKA are well-elucidated mechanisms [30,31,32,34]. These mechanisms are potentially linked and could enhance TRPA1 activity and/or trafficking to the membrane, allowing the amplification of pain symptoms [32]. We found that inhibition of the PLC/PKC and PKA pathways attenuated the overt nociceptive behavior and the sensitizing effect on pain symptoms induced by a TRPA1 agonist in anastrozole-treated animals. Our findings suggest that PLC/PKC and PKA may act downstream from B_2_R and B_1_R activation to sensitize the TRPA1 channel, leading to painful anastrozole-induced symptoms. Since anastrozole action is peripherally restricted [79] and the agonists of the B_2_R, B_1_R, and TRPA1 channels and PLC/PKC and PKA signaling pathways inhibitors were locally administered, it is plausible that all the painful symptoms observed in this study are due to peripheral effects.

We reported that the B_2_R and B_1_R activation-dependent PLC/PKC and PKA intracellular signaling pathways seem to interact to sensitize the TRPA1 channel in anastrozole-induced pain. Thus, direct TRPA1 activation by anastrozole and indirect TRPA1 stimulation from B_2_R and B_1_R activation appear to converge in a common nociceptive pathway. The interaction between kinin receptors and the TRPA1 channel suggests a novel paradigm to explain the development of the pain symptoms induced by anastrozole. Therefore, regulating the activation of signaling pathways downstream of B_2_R and B_1_R would be a promising alternative for developing drugs to treat AIs-related pain, such as anastrozole. The prompt and targeted treatment of pain symptoms might contribute to (i) re-establishing the quality of life of breast cancer survivors, (ii) improving adherence to AI therapy, and (iii) consequently contributing to disease control.

## Data Availability

Not applicable.

## Supplementary Materials

The following supporting information can be downloaded at: https://www.mdpi.com/article/10.3390/pharmaceutics15041136/s1. Figure S1: The B_2_R, B_1_R, and TRPA1 channels contribute to the development of painful behaviors induced by anastrozole in mice.

## Abbreviations

AI: Aromatase inhibitor; AITC, Allyl isothiocyanate; ANS, Anastrozole; B_1_R, Kinin B_1_ receptor; B_2_R, Kinin B_2_ receptor; Bk, bradykinin; BL, Baseline; Ca^2+^, calcium; CMC, Carboxy methylcellulose; DABk, Des-Arg^9^-bradykinin; DAG, Diacylglycerol; DALBk, Des-Arg^9^-[Leu^8^]-bradykinin; i.p., intraperitoneal; i.pl., intraplantar; Icat, Icatibant; Imax, Maximum inhibition; p.o., oral route; PWT, Paw Withdrawal Threshold; SEM, Standard Error Mean; TRP, Transient Receptor Potential; TRPA1, Transient Receptor Potential Ankyrin 1; Veh, Vehicle; A96, A967079; GF109, GF109203X; U73, U73122.
