# Supplementary material for: Kinin B2 and B1 Receptors Activation Sensitize the TRPA1 Channel Contributing to Anastrozole-Induced Pain Symptoms
Source: Pharmaceutics. 2023 Apr 3;15(4):1136. doi: 10.3390/pharmaceutics15041136 (PMC10143169; doi:10.3390/pharmaceutics15041136)
Supplement: Supplementary file 1 [file pharmaceutics-15-01136-s001.zip › pharmaceutics-2223299-supplementary.pdf]

Type of the Paper: Research Article

Supplementary Material

Kinin B_2 and B_1 receptors activation sensitize the TRPA1 channel contributing to anastrozole-induced pain symptoms

Maria Fernanda Pessano Fialho [1], Evelyne Silva Brum [1], Gabriela Becker [1], Indiara Brusco [1], Sara Marchesan Oliveira [1,2,*]

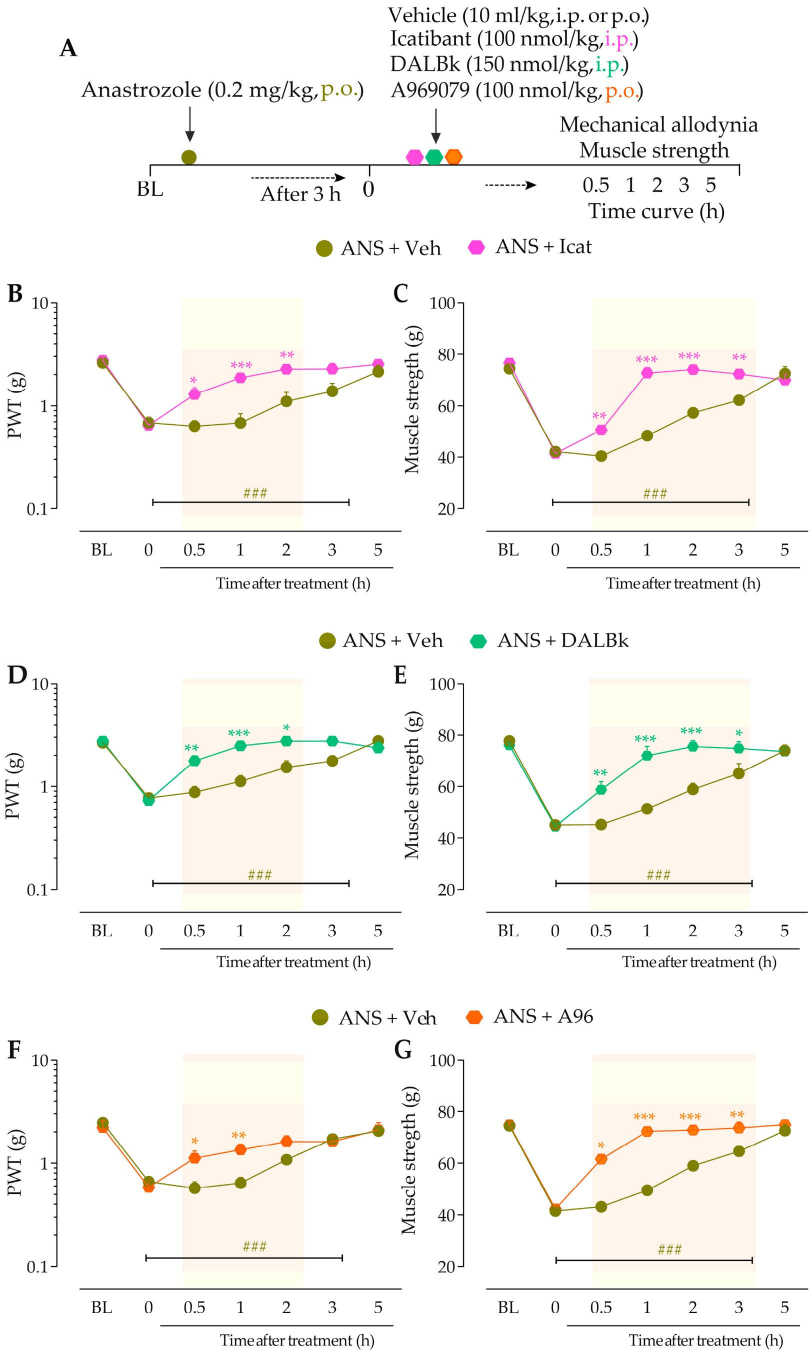

Supplementary Figure S1. The B_2R, B_1R, and TRPA1 channels contribute to the development of painful behaviours induced by anastrozole in mice. (A) Male C57BL/6 mice treated by oral route (p.o.) with anastrozole (0.2 mg/kg) presented mechanical allodynia and muscle strength loss compared to its vehicle (0.5% CMC). At 3 h after anastrozole administration (Time 0), the animals received a single administration of Icatibant (100 nmol/kg, intraperitoneal, i.p., B_2R antagonist), DALBk (150 nmol/kg, i.p., B_1R antagonist), A967079 (100 mg/kg, p.o., TRPA1 antagonist), or their vehicles (10 ml/kg, i.p. and p.o.). The PWT (B, D, and F) and strength muscle (C, E, and G) were evaluated again from 0.5 up to 5 h after antagonists' treatment. Baseline (BL) values were measured before anastrozole or vehicle administration. #p<0.05, ##p<0.01 and ###p<0.001 vs. BL values. *p<0.05, **p<0.01, and ***p<0.001 vs. ANS plus vehicle group. Data were expressed as the mean + SEM (n=6/group) and analyzed by two-way ANOVA followed by the Bonferroni post hoc test. Veh: intraperitoneal or oral vehicle injections; ANS: anastrozole oral administration; Icat: Icatibant intraperitoneal treatment; A96: A967079 oral treatment; PWT: paw withdrawal threshold.
